# Supplementary material for: Structural Insights into the Phosphorylation-Enhanced Deubiquitinating Activity of UCHL3 and Ubiquitin Chain Cleavage Preference Analysis
Source: Int J Mol Sci. 2022 Sep 15;23(18):10789. doi: 10.3390/ijms231810789 (PMC9501053; doi:10.3390/ijms231810789)
Supplement: Supplementary file 1 [file ijms-23-10789-s001.zip › ijms-1886978-supplementary.pdf]

Structural Insights into the Phosphorylation-Enhanced Deubiquitinating Activity of UCHL3 and Ubiquitin Chain Cleavage Preference Analysis

Yujing Ren †, Beiming Yu †, Lihui Zhou, Feng Wang * and Yanfeng Wang *

Key Laboratory of Molecular Medicine and Biotherapy, School of Life Science, Beijing Institute of Technology, Beijing 100081, China
* Correspondence: yf@bit.edu.cn (Y.W.); wfeng@bit.edu.cn (F.W.)
† These authors contributed equally to this work.

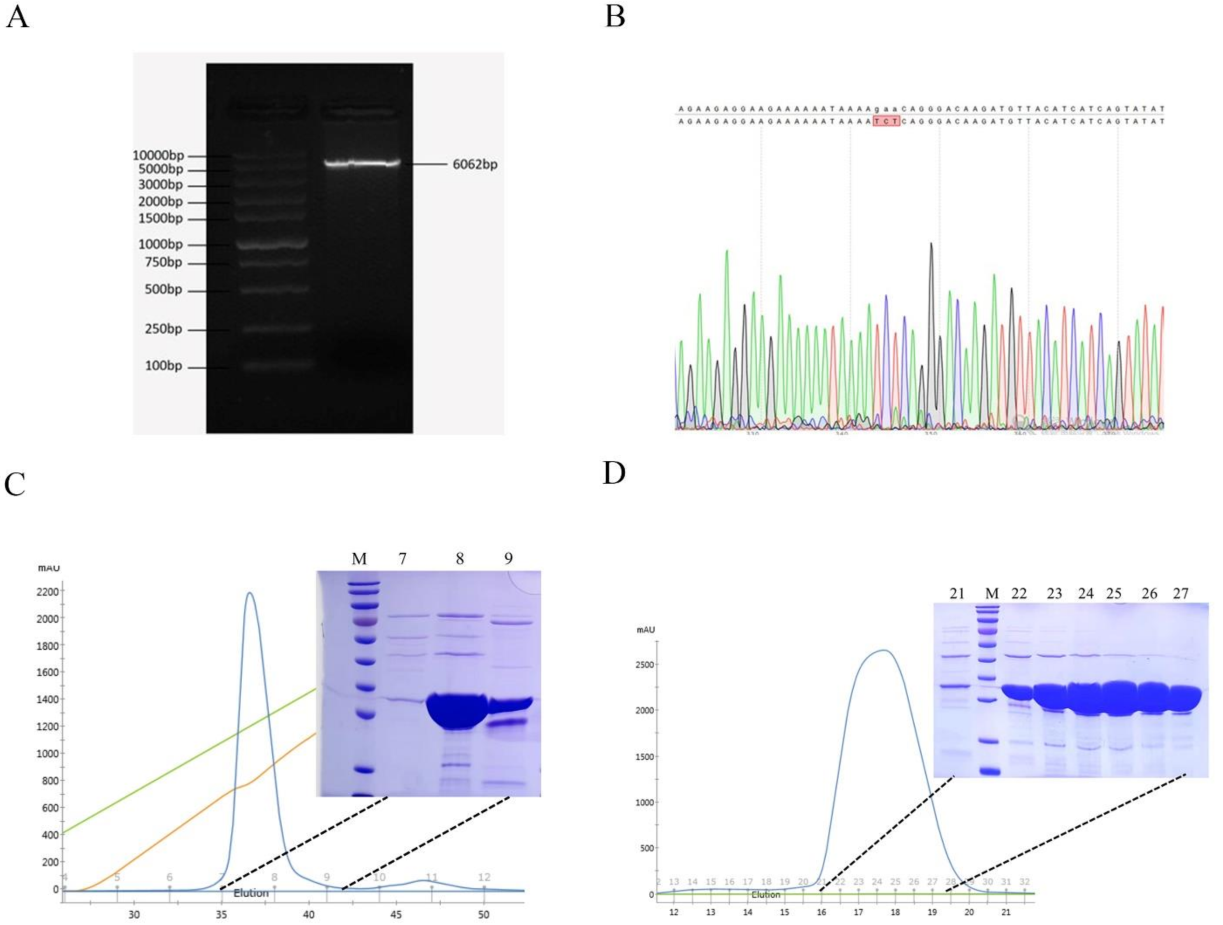

Figure S1. Plasmid construction and protein extraction and purification of ubiquitin C-terminal hydrolase-L3 (UCHL3) ^{S75E}. (A) The construction of UCHL3^{S75E}, result showed that plasmid of pET28a-UCHL3^{S75E} was successfully constructed. (B) The sequencing of UCHL3^{S75E}, result showed that Ser75 was successfully replaced Glu75. (C-D) The purfication of UCHL3^{S75E} by Hitrap Q(GE) and Superdex200(GE) respectively, and peak fractions were analyzed by SDS-PAGE and Coomassie blue staining. Results showed that the relatively pure UCHL3^{S75E} was obtained.

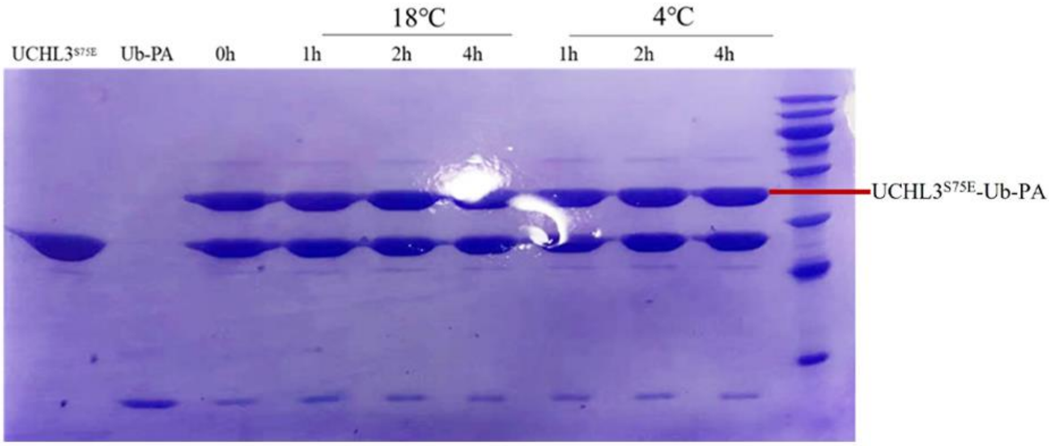

Figure S2. *In vitro* Ub-PA assay. The Ub-PA assay showed that the binding of Ub-PA to UCHL3^{S75E}: UCHL3^{S75E} was incubated with Ub-PA in a molar ratio of 2:1 in a 100ul reaction system contained 10 mM Tris-HCl, pH 7.5, 150 mM NaCl at 4 ℃ and 18 ℃ respectively, and samples were taken at 1h, 2h and 4h respectively. All the results were visualized by SDS-PAGE and Coomassie blue staining. Ub-PA ubiquitin-propargylamide.

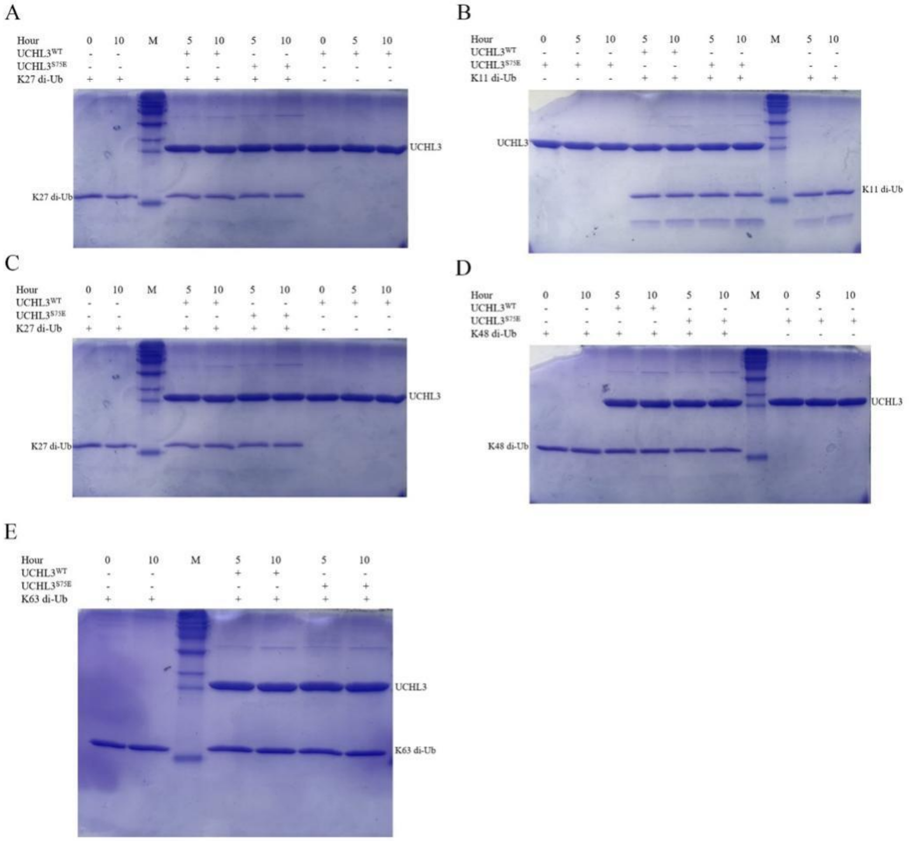

Figure S3. Raw data of the hydrolysis activity of UCHL3 toward different types of di-Ub. (A-E) Complete SDS/PAGE gel of the results of hydrolysis to di-Ub by UCHL3.

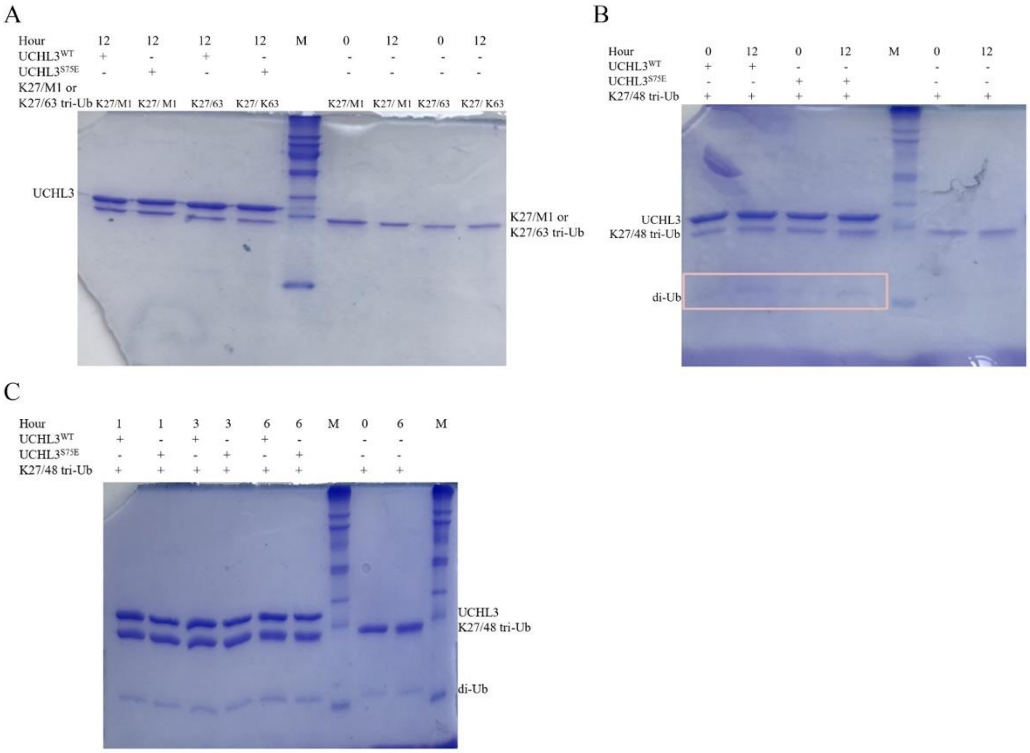

Figure S4. Raw data of the hydrolysis activity of UCHL3 toward different types of tir-Ub chains. (A-C) Complete SDS/PAGE gel of the results of hydrolysis to tri-Ub by UCHL3.
